# Effect of oral administration of soy-derived phosphatidic acid on concentrations of phosphatidic acid and lyso-phosphatidic acid molecular species in human plasma

**DOI:** 10.1186/1550-2783-10-S1-P22

**Published:** 2013-12-06

**Authors:** Martin Purpura, Ralf Jäger, Jordan M Joy, Ryan P Lowery, Jeff D Moore, Jacob M Wilson

**Affiliations:** 1 Increnovo LLC, 2138 E Lafayette Pl, Milwaukee, WI; USA; 2 Department of Health Sciences and Human Performance, The University of Tampa, Tampa, FL; USA; 3 Avanti Polar Lipids, Inc., 700 Industrial Park Drive, Alabaster, AL, USA

## Background

The glycerophospholipid Phosphatidic acid (PA) has been identified as a potential nutritional treatment for gastrointestinal disorders. Dietary food sources rich in PA include cabbage and radish leaves as well as *Mallotus japonicas*, a Japanese edible herb historically used for the treatment of stomach ulcers. The mammalian target of rapamycin (mTOR) has been shown to regulate rates of muscle protein synthesis and a mechanical stimulus (resistance exercise) has been shown to activate mTOR with PA playing a key role. Supplementation with soy-derived PA significantly increases responses in skeletal muscle hypertrophy, lean body mass, and maximal strength to resistance exercise. PA accounts for less than 0.1% of the total glycerophospholipid concentration of 201 mg/dl in the human plasma. 15 of the more than 600 distinct molecular lipid species quantified in human plasma are PA, 6 are lysophosphatidic acid (LPA). Orally administered PA can be metabolized to LPA and glycerophosphate by pancreatic phospholipases A1 and A2, which hydrolyze the fatty acid at the sn-1 position and the sn-2 position, respectively. Lysophospholipids are absorbed by the mucosal cells of the gastrointestinal tract and are rapidly re-acylated with fatty acids of the body pool resulting in a newly-formed phospholipid-molecule whose fatty acid composition is determined by the physiological and nutritional status and not by its source. This study sought to assess the effect of soy-derived PA supplementation on concentrations LPA and PA molecular species in human plasma.

## Methods

After a 12 hour overnight fast one subject (20 years of age, bodyweight of 82 kg, and height of 178 cm) was assigned to receive 1.5 grams of soy-derived PA (Mediator, Chemi Nutra, White Bear Lake, MN). Blood draws were taken immediately prior to, and at 30 min, 1, 2, 3, and 7 hours following supplementation. The samples were analyzed by an ultra-performance liquid chromatograph with triple quadrupole mass spectrometry (LC/MS/MS) using 17:1-LPA and 37:4-PA as internal standards to determine the concentration of LPA and PA molecular species in human plasma.

## Results

At baseline, 19 PA (highest concentrations: C34:2 (15%), C40:4 (11%), and C36:4 (10%)) and 5 LPA (16:0 (45%), 18:2 (19%), 20:4 (17%), 14:0 (11%) and 18:1 (8%)) molecular species could be quantified with total concentrations of PA of 2.66 nmol/ml, and LPA of 0.11 nmol/ml. Plasma concentrations of PA peaked at 3 hours (+32%) after ingestion and stayed elevated even after 7 hours (+18%). LPA showed a bimodal absorption kinetic with peaks after 1 hour (+500%) and 3 hours (+264%), after almost dropping back to baseline levels after 2 hours. On an individual fatty acid level, most prominent was a 23-fold increase in 20:4-LPA after 1 hour compared to baseline. The increase in 20:4-LPA does not result from the administration of PA, since soy-derived PA does not contain any arachidonic acid (fatty acids distribution of soy-PA: 18:2 (66.1%), 18:1 (12.6%), 16:0 (11.7%), 18:3 (6.1%) and 18:0 (3.4%)). Absorption of soy-derived PA must yield glycerophosphate which is re-acylated with arachidonic acid.

## Conclusion

LPA and PA can be molecularly identified and measured. LPA, PA and LPA+PA plasma levels increase 30 min after ingestions, plateau at 1-3 hours and remain above baseline levels after 7 hours. This is the first case study showing that orally administered PA is bioavailable. Future research should repeat this case study with a larger n-size and include the analysis of omega 3 fatty acid-LPA molecular species.

